# Baller-Gerold syndrome: Further evidence for association with prenatal exposure to valproate

**DOI:** 10.4103/0972-2327.40228

**Published:** 2008

**Authors:** Mary lype, PY Henry, CS Aravind, K Arun

**Affiliations:** Department of Paediatric Neurology, Government Medical College, Trivandrum, India; * Department of Paediatric Surgery, Government Medical College, Trivandrum, India; ** Department of Orthopaedics, Government Medical College, Trivandrum, India

**Keywords:** Adverse reactions-serious, Baller - Gerold syndrome, teratogenesis, valproic-acid

## Abstract

Baller Gerold Syndrome (BGS) is a rare autosomal recessive disorder that is apparent at birth. The disorder is characterized by distinctive malformations of the skull and facial area and bones of the forearms and hands. We are reporting a new case of BGS in a 10-month-old female child born of an epileptic mother who was on sodium valproate during the initial months of pregnancy. The baby was born with premature closure of the metopic suture, unilateral radial aplasia with limb malformation and other congenital anomalies that conformed with the description of BGS. The parents and other family members were unaffected, karyotyping was normal and there was no history of consanguinity. Fetal valproate exposure has been previously reported as the cause of this fetal malformation syndrome, which is generally inherited as an autosomal recessive trait. The peculiar pregnancy history and the supporting literature on the effects of valproic acid on the fetus exposed *in utero* to it with numerous case reports in the literature referring to BGS as a result of fetal exposure to valproate made us conclude that this is indeed a case of BGS secondary to valproate-induced teratogenesis.

## Introduction

Sodium valproate is an older, first-generation antiepileptic drug (AED), licensed in 1978. The first serious adverse reaction in pregnancy was reported in 1980.[1] Since then the teratogenicity of this drug has been emphasized. Among the antiepileptic medications in use, sodium valproate appears to be associated with the highest risk of birth defects as doses exceed 1000 mg/day.[2] The factors recognized to compound the teratogenic effect of this drug include use of multiple drugs, the fetal age at drug exposure and the differences in the genetic constitution of the mother and fetus.[3] The fact that the serum level of valproic acid is higher in the fetus than in the mother at any point of time makes the issue more complicated. The major congenital anomalies linked to valproate are neural tube defects, congenital heart defects, oral clefts, genital abnormalities and limb defects. Supernumerary nipples, postaxial polydactyly, bifid ribs and preaxial defects of the feet are some of the minor drug-induced anomalies associated with valproate.[4]

In a case series by Lajeunie *et al*, 17 patients born to mothers who had received valproate monotherapy exhibited trigonocephaly and 8 of these patients had a variety of limb anomalies.[5] BGS is as a rule autosomal recessive with causal mutations demonstrated in the RECQL4 gene. No sex predilection has been reported.[6] There are several reports that link BGS to the use of sodium valproate in pregnancy.[7–10] This syndrome was originally reported in the medical literature in 1950 (Baller F) and 1959 (Gerold M). The major features of BGS reported in 100% of the cases in the literature are craniosynostosis and preaxial upper limb defects.[11] In addition, affected patients may have anal, urogenital, cardiac, central nervous system and vertebral defects. However, BGS has been mistakenly diagnosed in several disorders that share phenotypic features with the syndrome; including Fanconi anemia,[12] VACTERL association with vertebral, anal, tracheal, cardiac, esophageal, renal and limb anomalies, Rothmund-Thompson syndrome[13] and RAPADILINO syndrome.[14]

## Case Report

A female child was born of spontaneous vaginal delivery to nonconsanguinous parentage. The mother was a 23-year-old woman who was on sodium valproate for 3 years prior to delivery for a seizure disorder with the onset at 19 years. She was on of sodium valproate (1400 mg/day) at the commencement of pregnancy using a combination of immediate and delayed release forms. She was on 5 mg of folic acid daily from 4 months before the pregnancy. While administering sodium valproate, she had a focal seizure with a fall due to the accompanying loss of consciousness. She was 4 weeks pregnant at that time, and on advice from her treating doctor, from that date, she was on an increasing dose of carbamazepine and a decreasing dose of sodium valproate for the next 8 weeks. Thereafter, she was asked to continue 1000 mg carbamazepine alone. In the twelfth week of pregnancy, she stopped carbamazepine also without informing her physician and had a seizure with a fall, four days after stopping the medication. She was started on carbamazepine once again and was seizure-free since then.

**Figure 1:**
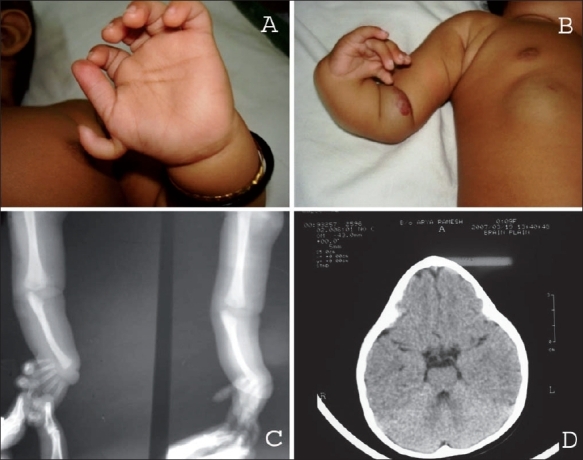
(A) Left hand showing an accessory thumb; (B) Right radial club hand and hemangiomas on the anterior aspect of the right elbow and the abdominal wall; (C) Anteroposterior and lateral view X-ray images of the right upper limb show the absent radius and the deformed short ulna in the right forearm; (D) CT scan of the brain showing normal brain parenchyma, fusion of the metopic suture with decrease in transverse diameter of the frontal region and anterior beaking in the mid frontal region (D). The sagital, the coronal and the lambdoid sutures are not fused

Serum alpha fetoprotein estimated at 18 weeks of pregnancy was normal and repeated fetal ultrasound scans done at 12, 18 and 20 weeks of pregnancy failed to pick up a congenital malformation. The pregnancy was uneventful thereafter and she continued her medication and had no seizure recurrence. A full-term female baby weighing 2.75kg (25^th^ centile), with a head circumference of 32.3 cm (5^th^ centile) and a length of 50cm (50^th^ centile) with multiple congenital anomalies was born [Figure 1]. The baby had a preaxial polydactyly on the left hand, a rudimentary right thumb, a hemangioma on the anterior aspect of the right elbow and on the right abdominal wall. There was flexion deformity of the right hand with malformed palm and fingers - radial club hand. X-ray images of the right forearm revealed the complete absence of the right radius with a short and deformed ulna [Figure 1C]. There was a prominent mid-frontal ridge and a triangular shape of the head when viewed from above the forehead. She had a slanting forehead, a mid face hypoplasia, a depressed nasal bridge, epicanthal folds and a long philtrum. The child presented at 10 months of age with only mild developmental delay. The baby attained social smile at 6 weeks of age, head steadiness by 3.5 months and sat with support at 6 months. She attained all the expected motor and mental milestones up to the twelfth month with a delay of not more than 14 days at any point of time. Her growth parameters at one year of age were normal with a weight of 9.5 kg (50^th^ centile); head circumference, 44.2 cm (25^th^ centile); and length, 74 cm (50^th^ centile). She had full utility of her left hand and had limited use of her right hand due to the deformity. CT scan of the head showed only premature fusion of the metopic suture [Figure 1D]. Karyotyping was normal. The patient underwent investigations to rule out vertebral anomalies and renal anomalies. She had an Ostium secundum atrial septal defect with a mild tricuspid regurgitation and no pulmonary arterial hypertension. She had no pancytopenia or thrombocytopenia. A routine metabolic screen for an inborn error of metabolism, including a urine metabolic screen, test for aminoaciduria, serum ammonia, lactate and pyruvate estimations were normal.

Table 1 lists the clinical features usually observed in fetal valproate syndrome compared to the phenotypic features of this child. It was concluded that the child's phenotype conformed to the described features of Baller-Gerold syndrome that is characterized by craniosynostosis, radial aplasia and multiple congenital anomalies. However, the prenatal exposure to valproate and the previous association of this syndrome with prenatal exposure of the fetus to valproate made us take this as yet another evidence for valproate as a cause for BGS. The surgery for craniosynostosis was not planned as the child's development was near normal and the parents were not keen on cosmetic surgery. The patient had undergone splinting and plaster-casting for the radial club hand with no benefit and has undergone surgical correction of the right limb deformity with excision of the accessory left thumb.

**Table 1:** Clinical features usually observed in fetal valproate syndrome and the features that are common to the described case

	Fetal valproate syndrome	The clinical features shared by the case reported
Major congenital malformations	Neural tube defects	Congenital heart defects
	Congenital heart defects	Limb defects
	Oral clefts and limb defects	
	Genital abnormalities	
Minor malformations	Inguinal and umbilical hernia,	Polydactyly
	Supernumerary nipples	
	Polydactyly, bifid ribs	
	Preaxial defect of feet	
Facial features	Trigonocephaly and metopic ridging	Trigonocephaly
	Tall forehead with bifrontal narrowing	Tall forehead with bifrontal narrowing
	Epicanthic folds, infraorbital groove	Epicanthic folds
	Medial deficiency of eyebrows	Flat nasal bridge
	Flat nasal bridge, broad nasal root	Broad nasal root
	Anteverted nares, shallow philtrum, Long upper lip and thin vermillion borders	Anteverted nares
	Thick lower lip. small downturned mouth	

## Discussion

The diagnosis of Baller-Gerold syndrome was entertained in this case after excluding hematopoetic abnormalities and additional malformations observed in other syndromes with limb anomalies and preaxial upper limb defects. A more detailed genetic analysis is lacking due to the lack of facility for the same.[12,13] The available literature does not contribute any phenotypic feature to distinguish genetically transmitted BGS from the same syndrome due to drug-induced teratogenesis. Sodium valproate has been consistently associated with long bone and preaxial deficiencies in infants exposed to the drug *in utero*, and the risk of limb anomalies with intrauterine exposure to the drug is estimated to be 0.42%.[7,8] Bifrontal narrowing and prominence of the metopic suture are characteristic of fetal valproate exposure.[8] A long philtrum, a thin upper lip, micrognathia, mid-face hypoplasia, epicanthal folds and a flat nasal bridge are also observed in infants exposed to sodium valproate *in utero*. In a recent case series, only one of the twins exposed to sodium valproate *in utero* was born with congenital anomalies that conformed to the described features of BGS;[15] the other twin did not have any congenital anomalies. This highlights the individual genetic susceptibility to teratogenicity. In radial club hand, there is an aplasia of the radius that causes the wrist to be in a fixed bent position toward the thumb side of the hand. There may be a deformity or absence of the thumb associated. This patient had the most severe form of radial club hand (type 4) that necessitates surgery for correction. This anomaly specifically develops early in pregnancy, sometime between the 28th and 56^th^ day of gestation when the bones of the hand and forearm are being formed. It has also been linked to some chromosomal abnormalities including trisomy 13, 18 and 21.

In this case even though the possibility of genetically mediated BGS is not entirely ruled out in the absence of a detailed genetic analysis, we have a strong case for teratogenesis since the baby was antenatally exposed to sodium valproate, carbamazepine and multiple seizures. An association to sodium valproate is high since there are previous such reports.[7–10,15] Nevertheless, the contribution of carbamazepine and seizures to teratogenesis in this case cannot be completely excluded.

The phenotypic features of this child resemble BGS, possibly associated with antenatal exposure to antiepileptic drugs and hypoxia contributed by recurrent seizures. Whether sodium valproate alone was the teratogenic factor or carbamazepine also contributed to the teratogenesis or the seizures also had a contributory role is open to debate. The fact that a combination of Sodium valproate and carbamazepine was used early in pregnancy is noteworthy since a baby with a major congenital malformation was born. This may be a pointer that this combination, even when low doses of carbamazepine is used, must be avoided at all costs in the pregnant epileptic. The high dose of sodium valproate at the commencement of pregnancy is also noteworthy, and this case may add further evidence to the well known fact that teratogenetic effects are observed more often in pregnant epileptics who are on higher doses of sodium valproate.[2]
